# Individual- and community-level determinants of child immunization in the Democratic Republic of Congo: A multilevel analysis

**DOI:** 10.1371/journal.pone.0202742

**Published:** 2018-08-23

**Authors:** Pawan Acharya, Hallgeir Kismul, Mala Ali Mapatano, Anne Hatløy

**Affiliations:** 1 Nepal Development Society, Bharatpur, Chitwan, Nepal; 2 Centre for International Health, University of Bergen, Bergen, Norway; 3 Department of Nutrition, School of Public Health, University of Kinshasa, Kinshasa, Democratic Republic of Congo; 4 Fafo, Institute for Labour and Social Research, Oslo, Norway; Johns Hopkins Bloomberg School of Public Health, UNITED STATES

## Abstract

Understanding modifiable determinants of full immunization of children provide a valuable contribution to immunization programs and help reduce disease, disability, and death. This study is aimed to assess the individual and community-level determinants of full immunization coverage among children in the Democratic Republic of Congo. This study used data from the Demographic and Health Survey 2013–14 from the Democratic Republic of Congo. Data regarding total 3,366 children between 12 and 23 months of age were used in this study. Children who were immunized with one dose of BCG, three doses of polio, three doses of DPT, and a dose of measles vaccine was considered fully immunized. Descriptive statistics were calculated for the prevalence and distribution of full immunization coverage. Two-level multilevel logistic regression analysis, with individual-level (level 1) characteristics nested within community-level (level 2) characteristics, was used to assess the individual- and community-level determinants of full immunization coverage. This study found that about 45.3% [95%CI: 42.02, 48.52] of children aged 12–23 months were fully immunized in the DRC. The results confirmed immunization coverage varied and ranged between 5.8% in Mongala province to 70.6% in Nord-Kivu province. Results from multilevel analysis revealed that, four Antenatal Care (ANC) visits [AOR: 1.64; 95%CI: 1.23, 2.18], institutional delivery [AOR: 2.37; 95%CI: 1.52, 3.72], and Postnatal Care (PNC) service utilization [AOR: 1.43; 95%CI: 1.04, 1.95] were statistically significantly associated with the full immunization coverage. Similarly, children of mothers with secondary or higher education [AOR: 1.32; 95%CI: 1.00, 1.81] and from the richest wealth quintile [AOR: 1.96; 95%CI: 1.18, 3.27] had significantly higher odds of being fully immunized compared to their counterparts whose mothers were relatively poorer and less educated. Among the community-level characteristics, residents of the community with a higher rate of institutional delivery [AOR: 2.36; 95%CI: 1.59, 3.51] were found to be positively associated with the full immunization coverage. Also, the random effect result found about 35% of the variation in immunization coverage among the communities was attributed to community-level factors.The Democratic Republic of Congo has a noteworthy gap in full immunization coverage. Modifiable factors–particularly health service utilization including four ANC visits, institutional delivery, and postnatal visits–had a strong positive effect on full immunization coverage. The study underlines the importance of promoting immunization programs tailored to the poor and women with little education.

## Background

Sub-Saharan Africa (SSA) has the world’s highest risk of neonatal deaths sharing 40% of the under-five death globally [1]. With an under-five mortality rate of 94 per 1000 live births, the Democratic Republic of Congo (DRC) has one of the highest child death rates in sub-Saharan Africa [2, 3]. Vaccine-preventable diseases such as tuberculosis and lower respiratory tract infection are still the leading causes of death in children in the DRC [4]. Childhood vaccination is, therefore, one of the most effective ways to reduce child mortality rates [5]. The World Health Organization (WHO) estimates that immunization averts 2 to 3 million deaths annually, and there is still scope to save an additional 1.5 million lives [6].

Global immunization coverage has increased in the past few decades [7, 8]. The Expanded Programme on Immunization (EPI) has played a central role in improving immunization coverage. The proportion of children receiving the vaccine against diphtheria, tetanus, and pertussis (DPT3) vaccine is typically used as a major indicator of the country’s capability of providing immunization services. In 2000, the global DTP3 coverage was 72%, and it increased to 86% by 2016 [9]. There has also been an improvement in measles vaccination. During the 1990s, measles coverage was about 71%, but since 2000 there has been a good increase, and in 2016, nearly 85% of children had received one dose of measles vaccine by their second birthday [9]. However, still, an estimated 19.4 million infants worldwide missed out on basic vaccines in 2015 [9]. About two-thirds of children without immunization coverage live in the DRC, Angola, Ethiopia, India, Indonesia, Iraq, Nigeria, Pakistan, the Philippines, and Ukraine [9].

Studies have shown that maternal education, socioeconomic status, and maternal service utilization during antenatal care (ANC), during delivery, and postnatal care associated with child immunization [10–14]. Additionally, factors such as media exposure, perceptions of vaccination, child’s place of birth (e.g. health facility), and region of residence also influence the immunization coverage among children [10–13]. Furthermore, the application of multilevel modeling has shown that community/contextual characteristics such as region or province of residence [12, 15, 16], community maternal education [15, 16], level of ANC utilization [12], level of the institutional delivery [15], and community poverty level [12] are important determinants of immunization service utilization. The DHS surveys are designed in such a way that the individual-level characteristics are nested within community-level (or primary sampling unit level) characteristics. Therefore, the multilevel analysis is a highly recommended tool while further analyzing such data. In fact, omitting the community-level characteristics while estimating the determinants of immunization coverage using the DHS data could bias the results [17].First DHS survey in the DRC in 2007 found that only 31% of 12–23-month-old children were fully immunized [18]. The Centers for Disease Control and Prevention (CDC) estimates that more than 764,400 children were unimmunized in the DRC in 2014 [19]. The gap in immunization coverage possesses a threat to the DRC’s global commitment to eliminate measles by 2020 [20]; therefore, some researchers have called for better EPI coverage and mass vaccination campaigns [21]. Similarly, previous local studies from Kinshasa and Goma in the DRC have examined factors determining child immunization. The studies have found that fullness and timeliness of immunization is determined by various factors such as the type of clinic in which an infant is enrolled for immunization, socio-economic status, social and family support to mothers, maternal education, and marital status [22–24]. There is no national-level study examining factors with implications for full immunization coverage in the DRC till date. Therefore, there is a need to better understand the level of immunization and variables related to the prevalence of immunization at the national level.

This study aims to assess the individual- and community-level determinants of child immunization coverage in the DRC using nationally representative, population-based, demographic and health survey data from 2013–14.

## Material and methods

### Study area

The DRC is one of the Central African countries with extensive amounts of natural resources. The total area of the DRC is 2.3 million square kilometers which is administratively divided into 26 provinces. The estimated total population of the DRC was 77.8 million in 2012 out of which 70% lived in rural areas.

At the national level, EPI administers the immunization program. The organization produces five-year plans, and these plans estimate the vaccine needs, cold chain supplies, and equipment needed to operate the immunization program. The EPI works in coordination with local partners to quantify the needs of the health zones. This information is then shared with UNICEF, which purchases immunization supplies [25].

### The survey

A multistage cluster sampling method was used for the DHS survey (EDS-RDC II). At the first stage, the national territory was divided into twenty-six sample domains corresponding to the DRC’s provinces. For urban areas, neighborhoods of cities and towns were sampled, whereas in rural areas, villages and chiefdoms were sampled. The final sampling unit selected was the cluster (neighborhood or village) and a total of 540 clusters were randomly selected as primary sampling units (PSUs). Subsequently, a fixed number of households were chosen from each of the selected clusters based on the probability proportional to size technique. A total of 18,360 households (5,474 in urban areas in 161 clusters and 12,886 in rural areas in 379 clusters) was drawn. DHS follows standard sampling procedure and detailed information can be obtained from the Measure DHS webpage [26]. Country-specific information is elaborated in the final report [27], which can be downloaded from the DHS website [28].

### Participant selection and exclusion criteria

A total of 3,441 children (unweighted number), aged 12–23 months, from 535 primary sampling units (DHS did not collect data from 5 sampling units) were included in this analysis. After adjusting the sample weight and cluster sample design, the sample size was equivalent to 3,366. Mothers, who were interviewed, were between the age group 15–49 years.

### Study variables

#### Dependent variable

The dependent variable for this study is the full immunization coverage among children aged between 12 and 23 months. Information on immunization was collected from the children’s immunization cards and interviews with their mothers. Children who had already taken a dose of BCG, three doses of DPT, three doses of polio, and a dose of measles vaccine were considered fully immunized and rest were considered not fully immunized.

#### Individual-level characteristics

Child-specific characteristics such as sex, birth order, preceding birth interval, and pregnancy intention were included in the analysis. Similarly, mother/parent-specific characteristics included were mother’s age, father’s age, education, marital status, mother’s occupational status, mother’s autonomy, father’s occupational status, and household wealth status measured in wealth quintile. Mother’s autonomy represents the level of a woman’s participation in making household decisions such as spending money, purchasing household goods/property, visiting friends/relatives and her ability to make decisions regarding her health care. Autonomy was categorized as autonomous if the woman could decide solely or jointly with her husband on all the above-mentioned issues. The socioeconomic group variable wealth quintile was calculated from household assets using principal component analysis and was divided into five categories (poorest, poorer, middle, richer and richest), each comprising 20% of the population [29].

Sex of household head, family size, and religion were also included in the analysis. Utilization of health services, including four ANC visits, institutional delivery, and postnatal care visit was also considered for analysis.

#### Community-level characteristics

Primary sampling units (PSUs) were considered proxies for the community level. Place of residence, distance from the health facility, community poverty rate, community ANC utilization rate, community institutional delivery rate, community postnatal visit rate, community maternal education, community media exposure rate, and community maternal unemployment rate were used as community-level characteristics. Community poverty rate is the proportion of individuals within the community living in the bottom 40% of wealth quintiles (poorer and poorest quintile collectively). Regarding the distance from the health facility variable, women were asked if they have experienced any difficulties in obtaining the medical advice or seeking treatment because of the distance between their home and the health facility. Their response was categorized as either a “big problem” or “not a big problem”, however, no physical distance in meters was measured. Every community-level characteristic, except the place of residence and distance to a health facility, was dichotomized into high and low, based on the median value.

### Statistical analysis

The distribution of full immunization coverage, according to the children’s background characteristics was cross-tabulated and the association was measured using the chi-square test. DHS-assigned sample weight was incorporated throughout the analysis. Similarly, bivariate logistic regression was performed to assess the unadjusted association between full immunization coverage and individual- and community-level predictors. The statistical significance was determined at a significance level of .05 and 95% confidence interval (CI). The Independent variables that were statistically significantly associated with immunization coverage in bivariate analysis were considered eligible for multivariate multilevel regression analysis.

A two-level multilevel modeling technique was used, as the individual-level characteristics were nested within the community-level characteristics. Level 1 modeling determined the association between individual-level predictors and childhood full immunization, while level 2 modeling assessed community-level determinants of full immunization.

The multilevel analysis consisted of four regression models. The first model (model 1) is an empty model without any individual- or community-level variables. This model measured the variation among the communities (primary sampling units). Model 2 consists of individual-level characteristics that were significant (p< .05) in the bivariate model. A stepwise backward elimination method was used to restrict the model to variables significantly associated with the immunization coverage. Such significantly associated variable in the previous model was considered eligible for the adjustment in the final model. Similarly, community-level characteristics significantly associated (p< .05) with the outcome variable in the bivariate logistic regression analysis were entered in model 3. This model was also based on a stepwise backward elimination technique. In the final multivariate model (model 4), individual- and community-level characteristics statistically significant in models 2 or 3 were included in the analysis and the final model was determined using stepwise backward elimination technique.

Stata’s “melogit” command was used for the multilevel logistic regression modeling to estimate fixed and random effect parameters. Fixed effects are measured as adjusted odds ratios and their 95% confidence intervals. Similarly, the random effects are the community-level (where the individual-level predictors are nested in) variance. The random effect is measured in the terms of Intra Community Correlation Coefficient (ICC) and Proportional Change in Community Level Variance (PCV). ICC is the measure of the percentage variance explained by the community-level variables, while PCV measures the proportional change in the community-level variance between the empty model and the subsequent models [30].

We also assessed two-way and three-way interaction terms; however, there was no statistically significant interaction within and between community- and individual-level predictors. Log-likelihood and Akaike’s Information Criterion (AIC) were used as model fit statistics.

## Results

About 45% of children aged between 12 and 23 months were fully immunized in the DRC. About 83% of the children were administered BCG vaccine. Polio coverage was found to be about 91% for the Polio 1, but it was reduced to 65.7% for the third dose (Polio 3). Similarly, about 72% of the children were vaccinated against measles. In the DRC, about 6% of the children aged 12–23 months were never vaccinated. Table 1 shows the coverage for the individual vaccine in the DRC. (Table 1)

**Table 1:** Status of child immunization coverage in the DRC in 2013–14 (*n* = 3,366).

Immunization	Coverage %	[95%CI]	
**BCG**	83.4	[81.26	85.61]
**DPT 1**	81.3	[78.88	83.62]
**DPT 2**	73.9	[70.86	76.83]
**DPT 3**	60.6	[57.27	63.83]
Polio 0*	49.9	[46.57	53.15]
**Polio 1**	91.7	[90.13	93.19]
**Polio 2**	84.5	[82.46	86.46]
**Polio 3**	65.7	[62.68	68.61]
**Measles**	71.6	[69.16	74.07]
**Full immunization**	45.3	[42.01	48.52]
**No vaccination**	5.9	[4.65	7.26]

*Oral polio vaccine (OPV) administered at birth and should be followed by the primary series of 3 OPV doses.

CI: Confidence interval.

Regarding the participant characteristics, there were almost equal numbers of male and female children. Four in every five children were born in a health facility. The same proportion of children had a sibling less than 24 month older than themselves. About 19% of children were born to teenage mothers. About 40% of mothers had a minimum of secondary level education and about 15.2% of the children were from unmarried or single mothers. Four of every five children were from households headed by men. (Table 2)

**Table 2:** Immunization coverage among 12–23-month-old children according to individual-level background characteristics in the DRC in 2013–14 (*n* = 3,366).

Characteristics	Total Sample	Full immunization coverage			
** **	***n***	**%**	**%**	**95% CI**	** **
**Sex of child**	** **	** **	** **	** **	***p* = .056**
Female	1679	49.9	45.5	[42.26,48.71]	
Male	1687	50.1	45.1	[41.82,48.36]	
**Birth order**		** **	** **	** **	***p* = .433**
1	646	19.2	49.2	[44.13,55.29]	
2	585	17.4	44.9	[38.38,51.66]	
3	501	14.9	42.7	[36.37,49.28]	
4	455	13.5	46.2	[39.71,52.76]	
5+	1179	35.0	44.0	[39.67,48.42]	
**Place of delivery**		** **	** **	** **	***p*< .001**
Home	701	20.8	20.8	[17.13,25.08]	
Health facility	2665	79.2	51.7	[49.09,54.29]	
**Preceding birth space<24 months**		** **	** **	** **	***p* = .026**
>24 months	2726	81.0	46.5	[43.97,49.08]	
<24 months	640	19.0	39.9	[34.94,45.15]	
**Pregnancy intention**		** **	** **	** **	***p* = .506**
Intended	2231	66.3	44.7	[41.94,47.49]	
Unintended	1135	33.7	46.4	[42.33,50.46]	
**Mother’s age at delivery**		** **	** **	** **	***p* = .941**
<20 years	647	19.2	45.8	[40.61,51.11]	
20–34 years	2255	67.0	45.3	[42.50,48.11]	
> = 35 years	464	13.8	44.4	[38.36,50.57]	
**Father’s age**		** **	** **	** **	***p*< .001**
<25 years	179	5.3	40.2	[30.60,50.55]	
25–34 years	1193	35.5	44.9	[41.04,48.80]	
> = 35 years	1699	50.5	53.9	[50.71,57.10]	
Missing	295	8.8			
**Mother’s education**		** **	** **	** **	***p*< .001**
Below secondary	2035	60.5	39.0	[36.20,41.96]	
Secondary or higher	1331	39.5	54.8	[51.09,58.43]	
**Father’s education**		** **	** **	** **	***p* = .107**
Below secondary	1158	34.4	42.7	[38.97,46.50]	
Secondary or higher	2208	65.6	46.6	[43.75,49.50]	
**Sex of household head**		** **	** **	** **	***p* = .126**
Male	2666	79.2	44.5	[41.85,47.08]	
Female	700	20.8	48.8	[43.90,53.72]	
**Marital Status**		** **	** **	** **	***p* = .608**
Unmarried/single	512	15.2	46.7	[40.82,52.66]	
Married or living with partner	2854	84.8	45.0	[42.53,47.51]	
**4ANC visit**		** **	** **	** **	***p*< .001**
No or <3 visits	1814	53.88	38.6	[35.58,41.73]	
4 or more	1552	46.12	53.0	[49.66,56.40]	
**TT before childbirth**		** **	** **	** **	***p* = .036**
No	2409	71.6	43.7	[41.03,46.39]	
Yes	957	28.4	49.2	[44.81,53.65]	
**Postnatal visit**		** **	** **	** **	***p* < .001**
No	2803	83.3	43.1	[40.61,45.58]	
PNC within 2 months	563	16.7	56.1	[50.31,61.81]	
**Religion**		** **	** **	** **	***p* = .060**
Catholic	898	26.7	47.7	[43.17,52.33]	
Protestant	980	29.1	44.0	[39.68,48.49]	
Other Christians	1234	36.7	46.4	[42.75,50.17]	
Other minorities	255	7.6	35.6	[29.11,42.69]	
**Mothers occupation**		** **	** **	** **	***p* = .003**
Not working	645	19.2	51.1	[45.70,56.40]	
Professional/technical/managerial	981	29.1	48.2	[43.98,52.51]	
Agriculture/unskilled/manual	1741	51.7	41.5	[38.33,44.64]	
**Fathers occupation**		** **	** **	** **	***p*< .001**
Not working	62	1.8	56.9	[37.02,74.82]	
Professional/technical/managerial	1505	44.7	50.1	[46.74,53.50]	
Agriculture/unskilled/manual	1619	48.1	39.6	[36.35,42.98]	
Missing	181	5.4	51.5	[42.00,60.81]	
**Family size**		** **	** **	** **	***p* = .320**
Small (<4 members)	772	22.9	43.7	[39.00,48.50]	
Medium (4–6 members)	1020	30.3	47.9	[43.67,52.23]	
Large (>8 members)	1574	46.8	44.3	[41.05,47.62]	
**Exposure to mass media**		** **	** **	** **	***p*< .001**
No	1858	55.2	38.9	[35.99,41.84]	
Some exposure	1508	44.8	53.1	[49.57,56.67]	
**Wealth quintile**		** **	** **	** **	***p*< .001**
Poorest	748	22.2	36.1	[31.61,40.82]	
Poorer	741	22.0	36.4	[31.57,41.56]	
Middle	645	19.2	43.7	[38.59,48.99]	
Richer	636	18.9	49.5	[44.11,54.80]	
Richest	596	17.7	65.0	[59.71,69.92]	
**Women’s autonomy**		** **	** **	** **	***p* = .029**
No autonomy	2570	76.3	43.9	[41.23,46.49]	
Autonomous	796	23.7	49.9	[45.16,54.54]	
Total (n = 3366)	3366	100.0	45.3	[42.98,47.57]	

CI: Confidence interval. *p-*values represent the results of weighted data.

The sex difference in immunization coverage among the children was not significant. According to the place of delivery, only one among every five children born at home was fully immunized, whereas every second child born in a health facility was fully immunized. The percentage of full immunization coverage was higher among the children of mothers who had 4 ANC visits, gave birth in a health facility, and made postnatal visits. Similarly, the percentage of children being fully immunized was found higher in upper wealth quintiles in comparison to lower quintiles. The coverage ranges from 36.1% among the poorest to 65% among the richest wealth quintile. The proportion of fully immunized children were higher if the mothers had autonomy in household decision making or if they had mass media exposure. (Table 2)

Regarding community-level characteristics, about two-thirds of the children were from rural areas. Distance to the health facility was a challenge to access the health care for about two in every five (41.2%) of the mothers. Similarly, about 45% of the children were from the communities with low ANC utilization. On the contrary, a majority (69.3%) of the children were from communities with high institutional delivery rates. (Table 3)

**Table 3:** Immunization coverage among 12–23-month-old children according to their contextual (community-level) background characteristics in the DRC in 2013–14 (*n* = 3,366).

Characteristics	Sample	Full immunization coverage			
	*n*	%	%	95% CI	
**Place of residence**	** **	** **	** **	** **	***p*< .001**
Rural	2283	67.8	41.6	[38.74,44.52]	
Urban	1083	32.2	53.0	[49.26,56.69]	
**Distance from the health facility**		** **	** **	** **	***p*< .001**
Big problem	1387	41.2	39.2	[35.88,42.69]	
Not a big problem	1979	58.8	49.5	[46.42,52.58]	
**Community mass media**		** **	** **	** **	***p*< .001**
Low	1649	48.98	38.1	[35.06,41.33]	
High	1717	51.02	52.1	[48.80,55.39]	
**Community poverty**		** **	** **	** **	***p*< .001**
Low	1723	51.2	53.6	[50.41,56.65]	
High	1643	48.8	36.6	[33.35,39.94]	
**Community maternal ANC utilization**		** **	** **	** **	***p*< .001**
Low	1525	45.3	39.6	[36.40,42.96]	
High	1841	54.7	49.9	[46.73,53.13]	
**Community institutional delivery**		** **	** **	** **	***p*< .001**
Low	1034	30.7	25.9	[22.37,29.71]	
High	2332	69.3	53.9	[51.07,56.64]	
**Community maternal unemployment**		** **	** **	** **	***P*< .001**
Low	1982	58.9	42.0	[39.04,45.02]	
High	1384	41.1	49.9	[46.37,53.50]	
**Community maternal education**		** **	** **	** **	***p*< .001**
Low	1746	51.9	39.1	[36.18,42.03]	
High	1620	48.1	52.0	[48.42,55.46]	

*p-* values represent the results from weighted data. CI: Confidence interval

Full immunization coverage was slightly lower in rural areas compared to urban areas (41.6% vs 53%). Similarly, about 40% of children from areas with low ANC utilization and about 50% of the high ANC utilization area were fully immunized. According to the institutional delivery utilization, a quarter of children from low coverage areas (25.9%) and more than half (53.9%) of children from high coverage areas were fully immunized. Similarly, about 39% of the children from the communities with lower maternal education rates were fully immunized, whereas 52% of the children from the communities with higher maternal education were found fully immunized. (Table 3)

**Fig 1:**
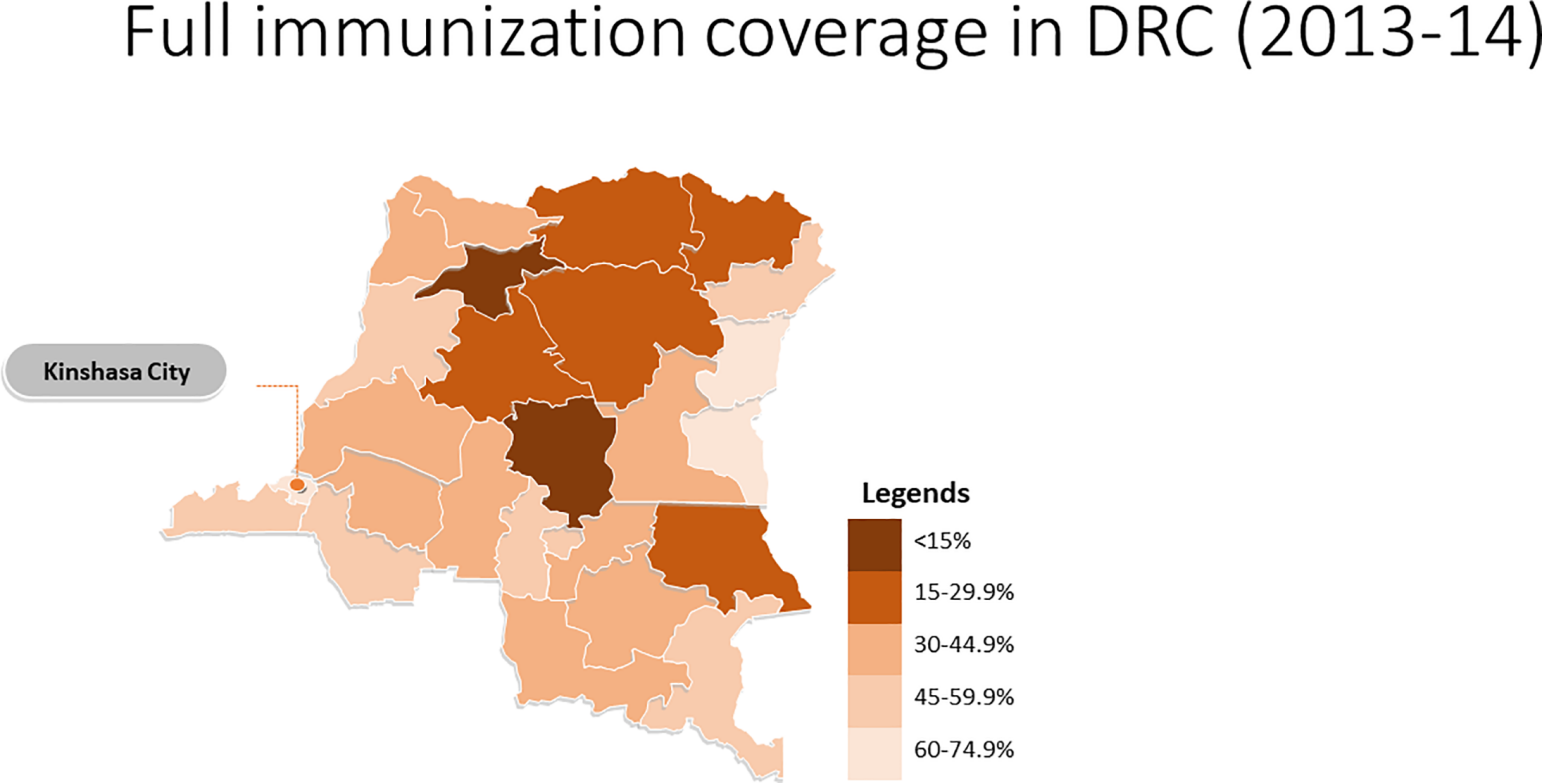
Full immunization coverage in DRC (2013–14).

Across the provinces, full immunization coverage ranged from 5.8% in Mongala to 70.6% in Nord-Kivu. The Nord-Kivu province was the only one which had higher coverage than the capital Kinshasa (67.7%). Fig 1, S1 Fig

Table 4 shows the results of the multilevel multivariate logistic regression analysis. The null model (Model 1) revealed significant variability on full immunization coverage across communities [τ = 1.82; p<0.001]. Similarly, the ICC showed that 50% of the variability in the odds of being fully immunized was due to community-level factors. (Table 4)

**Table 4:** Multivariate multilevel logistic regression analysis of individual and community level factors with childhood full immunization among 12–23-month-old children in the DRC in 2013–14 (*n* = 3,366).

	Model 1	Model 2	Model 3	Model 4
Characteristics	AOR(95%CI)	AOR(95%CI)	AOR(95%CI)	AOR(95%CI)
**Mother’s education**				
Below secondary		1.00		1.00
Secondary or higher		1.39 (1.02, 1.90)		1.32 (1.00, 1.81)
**ANC visits**				
No or <4		1.00		1.00
> = 4		1.67 (1.25, 2.23)		1.64 (1.23, 2.18)
**Place of delivery**				
Home		1.00		1.00
Health facility		3.07 (2.04, 4.60)		2.37 (1.52, 3.72)
**Postnatal care**				
No		1.00		1.00
Yes		1.44 (1.05, 1.970		1.43 (1.04, 1.95)
**Wealth status**				
Poorest		1.00		1.00
Poorer		0.92 (0.67, 1.26)		0.90 (0.65, 1.24)
Middle		1.03 (0.73, 1.47)		0.99 (0.70, 1.41)
Richer		1.50 (0.99, 2.29)		1.36 (0.89, 2.06)
Richest		2.44 (1.48, 4.03)		1.96 (1.18, 3.27)
**Community media exposure**				
Low			1.00	
High			1.37 (1.01, 1.86)	
**Community poverty**				
Low			1.00	
High			0.69 (0.49, 0.97)	
**Community institutional delivery**				
Low			1.00	1.00
High			3.95 (2.79, 5.58)	2.36 (1.59, 3.51)
**Intercept**	0.65 (0.55, 0.76)	0.12 (0.08, 0.18)	0.27 (0.18, 0.41)	0.11 (0.07, 0.16)
**Random effect**				
Community variance (SE)	1.82 (0.26)	1.40 (0.23)	1.29 (0.20)	1.34 (0.22)
PCV%		22.94	29.31	26.40
ICC%	50.12	37.37	33.43	35.25
**Model fit statistics**				
Log pseudolikelihood	-2046.58	-1936.55	-1987.64	-1926.94
AIC	4097.16	3895.09	3985.27	3875.89

AOR: Adjusted odds ratio; CI: Confidence interval. ICC: Intra-community correlation coefficient (ICC); PCV: Proportional change in community-level variance; AIC: Akaike’s information criterion. Model 1: Null/baseline model without any predictor variable. Model 2: Adjusted for individual-level predictor variables only. Model 3: Adjusted for community-level predictor variables only. Model 4: Adjusted for the individual- and community-level predictor variables.

After adjusting for individual-level characteristics, the variation in the odds of a child having full immunization remained statistically significant [τ = 1.40; p<0.001] across the communities. At the same time, about 37% of the variance in full immunization among the children was because of community-level factors. Similarly, about 23% of the variation in the odds of children being fully immunized between communities was attributed to the individual factors adjusted in the model (Model 2).

After adjusting the community-level characteristics, model 3 found a slightly reduced variance of a child being fully immunized [τ = 1.29] across the communities, as compared to the variance reported in model 2, and the variance also lost the statistical significance (p> .05). Model 3 further identified that about 33% of the variability in the odds of a child being fully vaccinated was due to community-level characteristics (ICC = 33.4%). Similarly, about 29% of the variability in the odds of children being fully immunized between communities could be explained by the community-level characteristics included in Model 3 (PCV = 29.3%).

Finally, after the statistical adjustment of the individual- and community-level characteristics simultaneously, Model 4 depicted significant variability across the communities with regards to the odds of a child being fully immunized [τ = 1.34; p<0.001]. About 35% of the variability in the odds of a child being fully vaccinated was due to the community-level factors (ICC = 35.3%). Similarly, the PCV revealed that about 26% of the variance in the odds of full immunization (PCV = 26.4%) across communities was due to the simultaneous effect of both individual and community-level characteristics adjusted in Model 4.

Furthermore, the final model found four ANC visits [AOR: 1.64; 95%CI: 1.23, 2.18], institutional delivery [AOR: 2.37; 95%CI: 1.52, 3.72], and postnatal care (PNC) service utilization [AOR: 1.43; 95%CI: 1.04, 1.95] were significantly associated with full immunization of children. Similarly, children of mothers with secondary or higher education [AOR: 1.32; 95%CI: 1.00, 1.81] and from the richest wealth quintile [AOR: 1.96; 95%CI: 1.18, 3.27] had significantly higher odds of being fully immunized compared to their counterparts. Among the community-level characteristics, a higher rate of institutional delivery [AOR: 2.36; 95%CI: 1.59, 3.51] was found to be positively associated with immunization coverage.

## Discussion

This study found a significant gap in immunization coverage in the DRC; only about 45% of children were fully immunized, an increase from 30.6% in 2007 [18]. The coverage of full immunization reported here was found to be higher than in some other sub-Saharan countries, including Ethiopia (24%) and Nigeria (25%) [31].

The study showed that there are significant regional differences in full immunization coverage. The results revealed that the coverage is especially poor in provinces such as Mongala and Sankuru. For example, Mongala, Sankuru, and Tshuapa provinces have poor health infrastructure, including a weak cold chain system, which is a major barrier to accessing the immunization program. Similarly, Tanganyika also has low vaccination coverage. Many local militias operate in this province. Political instability can therefore perhaps explain the province’s poor vaccination coverage. Despite the decades-long armed conflict, the immunization coverage in North and South Kivu is higher than in many other provinces. This might be because of the continuous effort of several organizations working in the immunization program. Our study is in line with other studies that have found regional differences in vaccine coverage [13]. While we have highlighted political factors that create geographical inequalities in vaccination coverage, other studies have highlighted factors such as cultural beliefs, health service capacity, modes of vaccine procurement, supply, and cold-chain management as determinants of immunization coverage [13, 25, 32].

The higher odds of being fully immunized found among children whose mothers did four ANC visits can be explained by the fact that ANC visits provide an opportunity to promote health care utilization, including institutional delivery, PNC, immunization, and family planning [33–35]. The association between ANC visits and child immunization is consistent with a study from India, which showed that ANC visits provide a platform for making mothers aware of child immunization [36]. This finding is consistent with several other studies [35, 37, 38].

Similarly, children born at a health facility were more likely to be fully immunized. The results corroborate with studies from Ethiopia [37], Uganda [39] Kenya [40], other SSA countries [35], and India [41, 42]. The finding that institutional delivery increased the chances of children being fully immunized can be explained by the fact that women who give birth at a facility are probably more likely to be aware of their own and their children’s health status. Women who utilized institutional delivery service, might also, be more confident in utilizing preventive services like child immunization. Also, administration of the BCG vaccine quickly after childbirth and vaccination counseling at a health facility might have contributed to the higher odds of full immunization.

This study found higher odds of being fully immunized when PNC was given by a skilled provider within two months after childbirth. A similar association between postnatal visits and full immunization coverage has been reported in several other studies, including a study of 14 LMICs [38], regional studies in Africa [31, 35] and a systematic review [43]. The association between PNC visits and immunization could be explained by the fact that an early postnatal visit provides an opportunity to initiate BCG vaccination. Also, DPT and polio vaccinations can be administered during PNC visits which could increase compliance with the immunization program and create an opportunity to initiate vaccination among children who are not immunized [12]. This study found that the ANC, delivery care, and PNC visits were independently associated with the full immunization coverage. A study from SSA countries reported similar findings [35]. An explanation for this might be that a continuum of care, with a four, recommended ANC visits, motivates pregnant women to give birth in a health facility. During the institutional delivery, a woman receives, not only the skilled care but also counseling and education to use the postnatal care and immunization services. ANC and institutional delivery open the door of opportunity for frequent contacts between health workers and a pregnant woman or recently delivered mothers, which eventually is more likely to result in higher compliance towards recommended immunization schedule [35].

This study showed that families with relatively higher wealth were more likely to fully immunize their children. Similar findings were published in studies from Nigeria [15, 31], Burkina Faso [44], Swaziland [45] and Ethiopia [12]. This finding was also consistent with a synthesis of DHS data in sub-Saharan Africa [16] and studies from South Asia, including Bangladesh [46] and India [41, 47]. The positive correlation between wealth and immunization can be explained by the fact that wealthier people tend to make better use of health services and thus regularly receive information about the benefit of child immunization [48]. On the other hand, high travel costs and long distances to health facilities can restrict poor people’s willingness to immunize their children [49].

Children from mothers with secondary or higher education had higher odds of being fully immunized. Similar findings were reported in studies from Ethiopia [50], Nigeria [31, 51], Kenya [40] and in India [47, 52]. Education was identified as a strong predictor of full immunization in children in several other studies [16, 37, 41, 42, 53]. Educated mothers are generally more aware of the importance of available health and immunization services, have better communication skills, and tend to better utilize available services [54, 55].

In regard to community characteristics, this study showed that there was a positive correlation between community institutional delivery and full immunization coverage. In line with this observation, research from Nigeria [15] and Ethiopia [50], also has found that children residing in communities with high institutional delivery service utilization were likely to be fully immunized.

### Significance and policy implications of this study

The link between full immunization coverage and child mortality has been well demonstrated by several researchers. A full immunization coverage as low as 45% suggests that children from the DRC are at high risk of avoidable death. A recent study by Doshi et al. showed that Congolese children are already at high risk of measles [56].

A landscape analysis of the DRC’s immunization program conducted by PATH provided major recommendations around supply chain management and raised awareness about the guiding policy, activities, and responsibilities across the national, provincial, and local health facility levels [25]. Similarly, UNICEF appealed for actions against the critical pitfalls of the DRC’s immunization program, including unreliability of government funding for vaccines, ineffective cold chain management, lack of proper support from health workers in certain regions and insufficient coordination of immunization stakeholders at provincial level [32].

This study demonstrates the links between ANC, institutional delivery, PNC visits and immunization coverage. Programs that make efforts to improve ANC utilization, institutional delivery rate, and PNC coverage should, therefore, draw attention to these links. Our study demonstrates the need to properly target vulnerable groups. Vaccination programs must pay special attention to women with low or no education. Similarly, programs also must direct their interventions for women and children living in poorer households. These modifiable determinants of full immunization could be integrated with supply-side factors to make a comprehensive package to improve immunization coverage in the DRC.

### Strengths, weaknesses, and limitations

This study identified important predictors of childhood full immunization in the DRC. It has described community-level variables in a multilevel model. Therefore, unlike studies with only individual characteristics, it has minimized bias due to omitted variables. Furthermore, the results are based on a nationally representative survey, making the findings generalizable with potentially important policy implications.

The study has some limitations. It is based on cross-sectional survey data, which makes it difficult to establish causal relationships between predictors and outcome variables. Data related to vaccination coverage is based on the records found in children’s immunization cards and interview with mothers. Therefore, some bias might have been introduced because of self-reporting. Also, because full immunization coverage (yes, no) is used as a binary outcome variable, children who were partially immunized were not analyzed separately, as it was outside the scope of this study, but were considered not fully immunized. Moreover, community-level characteristics were categorized as low or high based on the median value; hence, there was some loss of information as we dichotomized these continuous variables. Due to the retrospective nature of data, findings might be subject to recall bias. Furthermore, this study has not fully taken into consideration potential influential factors for immunization coverage, such as quality of service, counseling provided, follow-up and reminder communication, and distance to the immunization clinic.

## Conclusion

The DRC has a noteworthy gap in full immunization coverage. Individual-level characteristics,–particularly health services utilization, such as four ANC visits, institutional delivery, and postnatal visits–had a strong positive effect on full immunization coverage. Institutional delivery was also determined as a community-level factor. The findings suggest that maternal service sites might be effective in promoting full immunization coverage. Similarly, relatively higher wealth status and a minimum of secondary education had a positive effect on full immunization coverage. The study underlines the importance of promoting immunization programs tailored to the poor and women with little education. A major finding is a huge variation in immunization coverage across provinces. Efforts should be made to better understand the situation in provinces with low coverage.

### Ethical approval

Data from the Demographic and Health Survey, DRC (2013–14) was used for this study with the permission from the Measure DHS program. Ethical approval for the survey was obtained from the Ethics Committee of the School of Public Health (ESP) of the University of Kinshasa and the ICF International Institutional Review Board. Informed consent to participate in the survey was obtained verbally from each respondent before the interview was conducted. A special statement explaining the purpose of the study was included at the beginning of the household and the individual questionnaires. Participation in the survey was completely voluntary and the respondents were informed that they had the right to refuse to answer any questions or stop the interview at any point. The informed consent statement was read aloud exactly as it was written before the respondents were asked to participate in the interview. For participants under 18 years of age, verbal consent was obtained from their parent or legal guardian. After this, the interviewer signed his/her name attesting to the fact that he/she had read the consent statement to the respondent. However, given the low level of literacy and as the information requested was neither controversial nor sensitive; no written consent was obtained. The ethics committee of the School of Public Health of the University of Kinshasa and the ICF international Institutional Review Board waived the requirement for written consent of participants and approved the consent procedure used in this study.

## Supporting information

S1 Fig Level of full immunization coverage in DRC.pdf. (PDF)

## Abbreviations

BCG: Bacillus Calmette–Guérin
DTP: diphtheria, tetanus, and pertussis
DHS: Demographic and Health Survey
DRC: Democratic Republic of Congo
EPI: Expanded Program on Immunization
ICC: Intracommunity correlation coefficient (ICC)
PCV: proportional change in community-level variance
WHO: World Health Organization
