# Correction: Individual- and community-level determinants of child immunization in the Democratic Republic of Congo: A multilevel analysis

**DOI:** 10.1371/journal.pone.0211299

**Published:** 2019-01-17

**Authors:** Pawan Acharya, Hallgeir Kismul, Mala Ali Mapatano, Anne Hatløy

There are several errors in the headings for Tables 2 and 3. Please see the corrected Tables 2 and 3 here.

**Table 2:** Immunization coverage among 12–23-month-old children according to individual-level background characteristics in the DRC in 2013–14 (n = 3,366).

Characteristics	Children		Full immunization coverage		p-value
	*n*	%	%	95% CI	
**Sex of child**					
Female	1679	49.9	45.5	[42.26,48.71]	0.056
Male	1687	50.1	45.1	[41.82,48.36]	
**Birth order**					
1	646	19.2	49.2	[44.13,55.29]	0.433
2	585	17.4	44.9	[38.38,51.66]	
3	501	14.9	42.7	[36.37,49.28]	
4	455	13.5	46.2	[39.71,52.76]	
5+	1179	35.0	44.0	[39.67,48.42]	
**Place of delivery**					
Home	701	20.8	20.8	[17.13,25.08]	< 0.001
Health facility	2665	79.2	51.7	[49.09,54.29]	
**Preceding birth space<24 months**					
>24 months	2726	81.0	46.5	[43.97,49.08]	0.026
<24 months	640	19.0	39.9	[34.94,45.15]	
**Pregnancy intention**					
Intended	2231	66.3	44.7	[41.94,47.49]	0.506
Unintended	1135	33.7	46.4	[42.33,50.46]	
**Mother’s age at delivery**					
<20 years	647	19.2	45.8	[40.61,51.11]	0.941
20–34 years	2255	67.0	45.3	[42.50,48.11]	
> = 35 years	464	13.8	44.4	[38.36,50.57]	
**Father’s age**					
<25 years	179	5.3	40.2	[30.60,50.55]	< 0.001
25–34 years	1193	35.5	44.9	[41.04,48.80]	
> = 35 years	1699	50.5	53.9	[50.71,57.10]	
Missing	295	8.8			
**Mother’s education**					
Below secondary	2035	60.5	39.0	[36.20,41.96]	< 0.001
Secondary or higher	1331	39.5	54.8	[51.09,58.43]	
**Father’s education**					
Below secondary	1158	34.4	42.7	[38.97,46.50]	0.107
Secondary or higher	2208	65.6	46.6	[43.75,49.50]	
**Sex of household head**					
Male	2666	79.2	44.5	[41.85,47.08]	0.126
Female	700	20.8	48.8	[43.90,53.72]	
**Marital Status**					
Unmarried/single	512	15.2	46.7	[40.82,52.66]	0.608
Married or living with partner	2854	84.8	45.0	[42.53,47.51]	
**4ANC visit**					
No or <3 visits	1814	53.88	38.6	[35.58,41.73]	< 0.001
4 or more	1552	46.12	53.0	[49.66,56.40]	
**TT before childbirth**					
No	2409	71.6	43.7	[41.03,46.39]	0.036
Yes	957	28.4	49.2	[44.81,53.65]	
**Postnatal visit**					
No	2803	83.3	43.1	[40.61,45.58]	< 0.001
PNC within 2 months	563	16.7	56.1	[50.31,61.81]	
**Religion**					
Catholic	898	26.7	47.7	[43.17,52.33]	0.060
Protestant	980	29.1	44.0	[39.68,48.49]	
Other Christians	1234	36.7	46.4	[42.75,50.17]	
Other minorities	255	7.6	35.6	[29.11,42.69]	
**Mothers occupation**					
Not working	645	19.2	51.1	[45.70,56.40]	0.003
Professional/technical/managerial	981	29.1	48.2	[43.98,52.51]	
Agriculture/unskilled/manual	1741	51.7	41.5	[38.33,44.64]	
**Fathers occupation**					
Not working	62	1.8	56.9	[37.02,74.82]	0.001
Professional/technical/managerial	1505	44.7	50.1	[46.74,53.50]	
Agriculture/unskilled/manual	1619	48.1	39.6	[36.35,42.98]	
Missing	181	5.4	51.5	[42.00,60.81]	
**Family size**					
Small (<4 members)	772	22.9	43.7	[39.00,48.50]	0.320
Medium (4–6 members)	1020	30.3	47.9	[43.67,52.23]	
Large (>8 members)	1574	46.8	44.3	[41.05,47.62]	
**Exposure to mass media**					
No	1858	55.2	38.9	[35.99,41.84]	< 0.001
Some exposure	1508	44.8	53.1	[49.57,56.67]	
**Wealth quintile**					
Poorest	748	22.2	36.1	[31.61,40.82]	<0.001
Poorer	741	22.0	36.4	[31.57,41.56]	
Middle	645	19.2	43.7	[38.59,48.99]	
Richer	636	18.9	49.5	[44.11,54.80]	
Richest	596	17.7	65.0	[59.71,69.92]	
**Women’s autonomy**					
No autonomy	2570	76.3	43.9	[41.23,46.49]	0.029
Autonomous	796	23.7	49.9	[45.16,54.54]	
Total (n = 3366)	3366	100.0	45.3	[42.98,47.57]	

CI: Confidence interval

p-values represent the results of weighted data.

**Table 3:** Immunization coverage among 12–23-month-old children according to their contextual (community-level) background characteristics in the DRC in 2013–14 (n = 3,366).

Characteristics	Children		Full immunization coverage		
	*n*	%	%	95% CI	p-value
**Place of residence**					
Rural	2283	67.8	41.6	[38.74,44.52]	< 0.001
Urban	1083	32.2	53.0	[49.26,56.69]	
**Distance from the health facility**					
Big problem	1387	41.2	39.2	[35.88,42.69]	< 0.001
Not a big problem	1979	58.8	49.5	[46.42,52.58]	
**Community mass media**					
Low	1649	48.98	38.1	[35.06,41.33]	< 0.001
High	1717	51.02	52.1	[48.80,55.39]	
**Community poverty**					
Low	1723	51.2	53.6	[50.41,56.65]	< 0.001
High	1643	48.8	36.6	[33.35,39.94]	
**Community maternal ANC utilization**					
Low	1525	45.3	39.6	[36.40,42.96]	< 0.001
High	1841	54.7	49.9	[46.73,53.13]	
**Community institutional delivery**					
Low	1034	30.7	25.9	[22.37,29.71]	< 0.001
High	2332	69.3	53.9	[51.07,56.64]	
**Community maternal unemployment**					
Low	1982	58.9	42.0	[39.04,45.02]	< 0.001
High	1384	41.1	49.9	[46.37,53.50]	
**Community maternal education**					
Low	1746	51.9	39.1	[36.18,42.03]	< 0.001
High	1620	48.1	52.0	[48.42,55.46]	

*p-* values represent the results from weighted data

CI: Confidence interval
