# Supplementary figures and images for: Individual- and community-level determinants of child immunization in the Democratic Republic of Congo: A multilevel analysis
Source: PLoS One. 2018 Aug 23;13(8):e0202742. doi: 10.1371/journal.pone.0202742 (PMC6107214; doi:10.1371/journal.pone.0202742)

Level of full immunization coverage in DRC (2013-14)

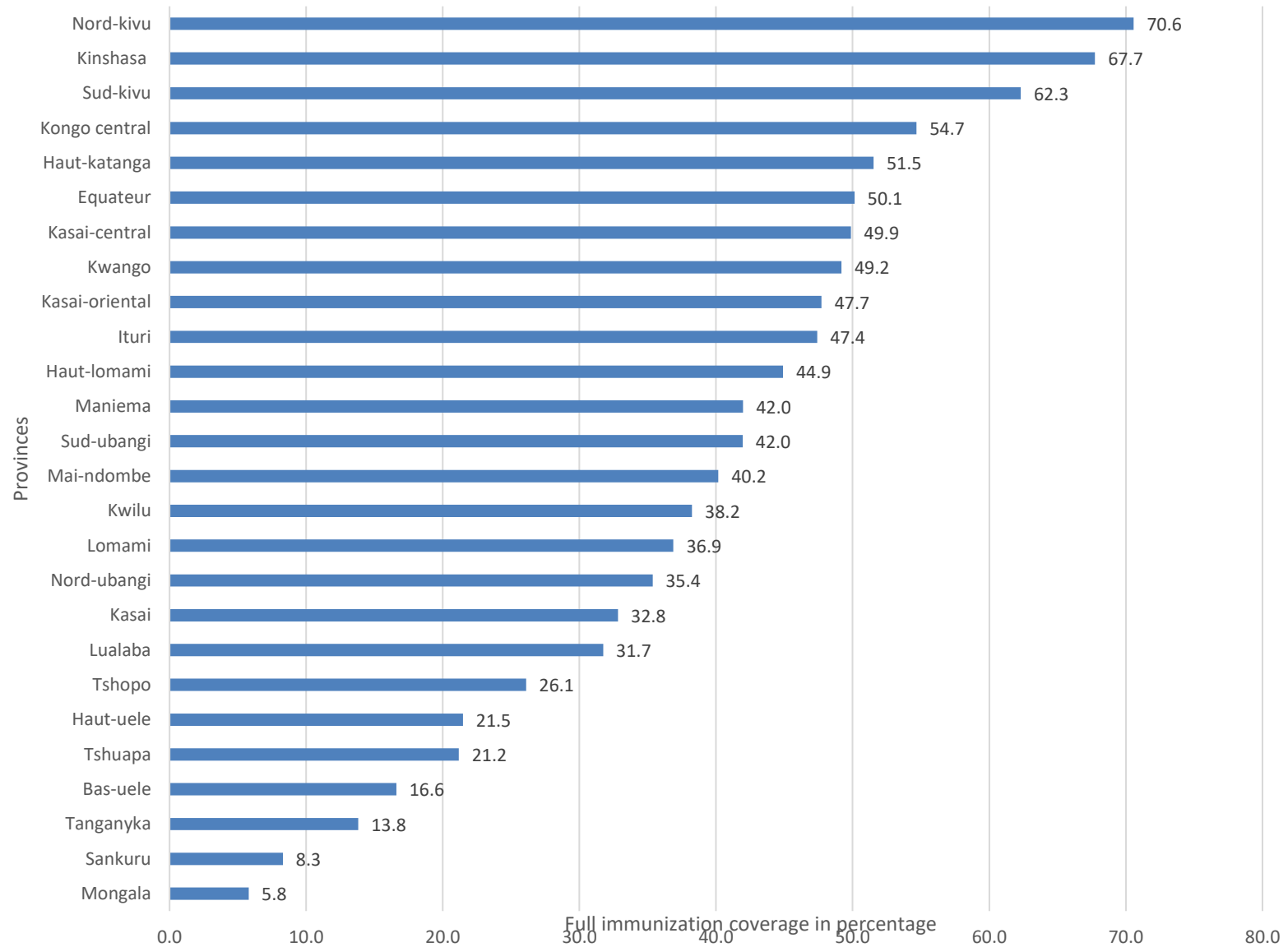

Supplement: S1 Fig — (PDF) [file pone.0202742.s001.pdf]
